# Speedometer and Consumeter

**Published:** 1909-08-14

**Authors:** 


### Motoring Notes.

### SPEEDOMETER AND CONSUMETER.

The value of a reliable speed indicator is apparent, and what no doubt weighs most with the average motorist is its value as evidence in police-court cases. It is well, therefore, when purchasing an instrument of this sort to choose an indicator which is regularly accepted as evidence by the magistrates. In this respect the position of Smith's Perfect Speed Indicator is unassailable, as the record of the week ending June 26 alone so clearly proves. In five cases during that week the Perfect Speed Indicator was called upon to give evidence, with the result that four of the summonses were dismissed. But apart from this very apparent value the speed indicator is useful in many ways. It enables the mere novice to become an expert in changing his speed at the correct moment, and it also informs him exactly how his engine is running by the speed obtained on the various gears. An entirely new use for the speed indicator, however, arises with the introduction of S. Smith and Son's consumeter or petrol-meter. This most indispensable accessory interposed in any position between the tank and the carburettor indicates (1) the total consumption in quarts and tenths of pints; (2) the actual consumption on any trip; (3) the amount in the tank. The latter two are recorded in gallons and quarters of a gallon, and are set from a small knob at the top of the case. It is, by means of a stop-watch, quite easy to ascertain at any moment the exact rate of consumption by taking the duration of time for any tenth of a pint; by means of a small slide-rule supplied with the instrument the consumption in pints per hour is shown, and by comparison with the speed per hour and a simple calculation the consumption in miles per gallon is arrived at. It is now, therefore, possible to take observations, if necessary, several times in one minute giving the actual ratio consumption of a car under all varying conditions of speed, load, and road gradients, and by this means to learn the actual power developed by the engine under these circumstances. The consumeter is not a toy, but an instrument of precision which enables a car-owner to know positively the most economic engine and road speeds for driving, to assure the correct proportions of air and petrol for the formation of a perfectly combustible mixture. Thus over-heating is avoided, and carbon deposits in the cylinders or the ejection of noisy and noxious gases in the exhaust are also obviated, while a check is kept upon the consumption and waste is prevented.

These two instruments, therefore, are worthy of every motorist's attention and consideration, and are really necessary if he wishes to bring up and maintain his car in a high state of mechanical efficiency.

